# Dressing patterns in healthy individuals and brain-damaged patients: the MIlan Dressing Assessment

**DOI:** 10.3389/fneur.2026.1852682

**Published:** 2026-08-12

**Authors:** Elisabetta Banco, Giacomo Guidali, Angela Rossetti, Irene Rossi, Carlotta Casati, Cecilia Chessa, Giuseppe Vallar, Nadia Bolognini

**Affiliations:** 1 Laboratory of Neuropsychology, Department of Neurorehabilitation Sciences, IRCCS Istituto Auxologico Italiano, Milan, Italy; 2 Department of Psychology & NeuroMI-Milan Center for Neuroscience, University of Milano-Bicocca, Milan, Italy; 3 O.U. Neuromotor Rehabilitation, Department of Neurorehabilitation Sciences, IRCCS Istituto Auxologico Italiano, Milan, Italy

**Keywords:** apraxia, assessment, brain-damaged patients, cognitive, dressing patterns, stroke

## Abstract

**Background:**

The ability to dress appropriately is a fundamental daily living skill that is often impaired by neurological disorders and can manifest as a specific form of apraxia (dressing apraxia). Despite its prevalence and impact on functional independence, standardized and reliable assessment tools for dressing skills remain limited. The aim of this study was to identify dressing patterns in healthy individuals and use them as a reference for introducing a new clinical tool to assess dressing skills.

**Methods:**

We conducted two studies, including seventy healthy participants (mean age = 48.73 ± 17.85 years; range = 20–87) and 22 brain-damaged patients (21 with stroke, 1 with a neoplastic lesion; mean age = 69.7 ± 13.1 years; range = 45–87). Dressing patterns for different garment types were identified among healthy participants and then used as a reference to assess dressing skills in brain-damaged patients using a 3-point rating scale across multiple garments, namely the MIlan Dressing Assessment (MIDA). Interrater reliability was evaluated using the intraclass correlation coefficient (ICC). Associations between dressing skills scores and demographic or clinical variables were assessed using Spearman correlations, while differences by lesion side and location, and neuropsychological deficits were examined using Mann–Whitney U tests.

**Results:**

Reliable dressing patterns were identified for eight garments in healthy participants; they were largely independent of demographic factors, though specific age-related variations emerged for five garments, and a sex-related difference was found for scarf wearing. Forty-five percent of brain-damaged patients presented with impaired ability to dress. In this clinical group, the MIDA demonstrated excellent inter-rater reliability (ICC = 0.93). Higher dressing skills scores were associated with lower neurological severity, greater functional independence, and better non-verbal reasoning skills. Dressing patterns differed in the presence of unilateral spatial neglect and constructional apraxia.

**Conclusion:**

The MIDA offers a novel structured framework to evaluate dressing skills and assess patients’ residual autonomy during this complex everyday activity. This exploratory study provides a foundation for future studies aimed at defining its broader feasibility and role in clinical practice.

## 1 Introduction

The ability to dress oneself requires performing a sequence of actions involving precise, independent yet coordinated movements of the upper and lower limbs. It is a fundamental activity of daily living, crucial for the functional independence of neurological patients. Consequently, dressing autonomy is an essential component included in various standard functional independence scales, including the Barthel Index (1) and the Functional Independence Measure [FIM; Keith et al. (2)].

Difficulties in dressing can be a primary symptom of apraxia. Apraxia refers to the inability to carry out skilled voluntary movements in the absence of motor, sensory, or elementary coordination deficits (3–5). Apraxia results from the disruption of the higher levels of gesture organization, those in which movement patterns consistent with the person’s intentions and appropriate to the environmental context are conceived and selected (6–8). This is one of the most common and disabling disorders caused by brain damage due to conditions like stroke, traumatic brain injury, or neurodegenerative disorders such as Alzheimer’s disease, Posterior Cortical Atrophy, or Parkinson’s disease. Limb apraxia is typically caused by left brain damage, with an incidence of about 50% or more, up to about 80%, depending on the type of apraxia. In neurodegenerative dementia, the incidence and range of limb apraxia are broadly comparable (9–12). Apraxia can be classified into subtypes such as ideomotor, ideational, and kinetic apraxia of the limbs, depending on the specific cognitive and processes of motor planning involved, or it can be distinguished based on the affected body parts; in this latter case we refer to buccofacial, trunk, and limb apraxia (13, 14), alongside other distinct forms such as constructional apraxia, that is, however, and independent disorder (15).

Beyond these classic neuropsychological taxonomies, clinical research has also identified task-specific variants, most notably dressing apraxia (13). Dressing apraxia was first described by the British neurologist W. Russell Brain (16), who reported two right-handed patients, one with a left (Case #1, head injury) and one with a right posterior parietal lesion (Case #4, tumor), who showed severe difficulties in dressing, making errors, such as putting clothes on back to front or upside down (16). Brain (16) termed this disorder “apraxia for dressing.” For both patients, no mention was made of other types of apraxia, and no constructional apraxia was observed. Case #4, however, had also left hemiplegia, homonymous hemianopia, and hemianaesthesia, which also may impair the ability to dress. Brain suggested that these dressing difficulties could be traced back to a defective body schema for one half of the body; patients showed symptoms such as impaired discrimination of the two sides of the body, and neglect of the contralateral side of the body (17).

Despite the impact of disorders of dressing on patients’ functional independence, their assessment and diagnosis have been mainly based on clinical observation, which is inherently descriptive, subjective, and non-systematic, and varies from patient to patient. There is a lack of standardized testing methods to assess dressing skills, in contrast to the availability of standardized methods of assessment for other apraxic disorders such as ideomotor [e.g., (18)], ideational (19), oral (20), and constructional apraxia (15). To evaluate these deficits, specific tests with normative data [e.g., the norms for an Italian sample of healthy participants provided by Spinnler and Tognoni (21)] have been developed and are routinely used in clinical practice (13).

A first attempt to develop a test to quantify dressing difficulties in neurological patients was made by Morera and colleagues (22), who investigated the ability of healthy individuals’ to put on and take off a cardigan. Van Heugten et al. (23, 24) made significant contributions to the standardization of observational methods for evaluating everyday activities, including dressing, specifically in stroke patients with apraxia (23, 24).

The only available tool for clinical practice is the Nottingham Stroke Dressing Assessment (NSDA) (25), which evaluates post-stroke dressing skills. The NSDA assesses independence across various dressing tasks (e.g., putting on and taking off different clothing items) using a standardized scoring system. Notably, this assessment does not specifically measure praxic skills for dressing; instead, it focuses on more general functional motor skills. More recently, research has shifted toward the psychometric validation of scales focused exclusively on upper-body dressing in stroke populations (26). However, these existing tools often remain limited to specific body parts or lack a direct, detailed comparison with data from healthy controls, thereby limiting the precision with which abnormal dressing patterns can be quantified.

The present study aimed at developing a new, specific tool going beyond a mere, non-specific evaluation of functional/motor autonomy in dressing. Unlike previous studies [e.g., (25, 27–29)], the ability to dress with garments suitable for different body parts (trunk, head, arms, legs, and feet) was also assessed. To this end, *Study #1,* performed in healthy participants, explored the main patterns of dressing and undressing for different clothing items, measured the time required to put on and take off various clothing items, and examined the possible effects of age on dressing. This allowed us to identify in healthy participants the main dressing patterns, namely, common fashions for putting on/take off clothes. Based on these data, we developed a protocol and scoring system for a standardized clinical evaluation of dressing disorders and performed an exploratory study in brain-damaged patients, to assess the protocol’s usability. To achieve this second aim, in the second experiment (*Study #2*), the task was administered to brain-damaged patients to assess its inter-rater reliability and preliminary associations with clinical measures, serving as an initial step toward the development of a new clinical instrument, named the “MIlan Dressing Assessment” (i.e., MIDA).

## 2 Materials and methods

Two studies were performed. In *Study #1*, a protocol for describing dressing and undressing patterns in healthy participants was developed. The aim was to identify possible ways of dressing in adults and to verify the prevalence of common dressing patterns (i.e., ways of putting on and taking off clothes), as well as the effects of age, educational level (i.e., years of schooling), and sex on these skills.

In *Study #2*, the protocol developed in healthy adults was administered to a group of neurological patients with acquired unilateral brain damage to assess their dressing/undressing patterns and potential dressing impairments.

All participants (healthy controls and brain-damaged patients) provided written informed consent to participate in the study, which included permission for video recording during the tasks. This consent was obtained from all individuals prior to the experimental session. Both studies were performed in accordance with the Declaration of Helsinki (2013) and approved by the Ethics Committee (protocol number 25C122) of the IRCCS Istituto Auxologico Italiano.

### 2.1 Study #1: identification of physiological patterns of dressing

#### 2.1.1 Participants

The first study involved 70 healthy participants, with no history or evidence of neurological and psychiatric disorders (mean age ± standard deviation – SD: 48.73 ± 17.85 years; range: 20–87 years; 40 females), who were recruited among acquaintances of the experimenters (E.B., A.R., C.C.) in northern Italy (Milan, Bergamo, Cremona).

#### 2.1.2 Experimental procedure

Participants were asked to put on and take off 10 garments suitable for the upper or lower body (cap, gloves, scarf, t-shirt, shirt, cardigan, zipper cardigan, slippers, bathrobe, trousers). Two items (shirt and cardigan) differed for men and women due to the different button placements.

During the assessment, the examiner asked each participant to wear each garment as naturally as possible, just as they would in everyday life. Once worn, each participant was asked to remove it. For the zipper cardigan, each participant was asked to zip and unzip it. All participants wore the same clothing items and were filmed throughout the dressing and undressing task.

The examiner administered the dressing assessment, which lasted about 20 min, and analyzed each video on a personal computer. A grid was created to report the dressing patterns that participants displayed for each garment. All patterns were described by dividing the wearing/removal process into several steps. To ensure high consistency in identifying these steps, the three examiners involved in administering the task (I.R., A.R., C.C.) underwent joint training sessions, analyzing a subset of videos together to calibrate and standardize the individual components of each dressing pattern. After this step, the different dressing schemes displayed by the healthy participants could be grouped into distinct behavioral categories, as identified and agreed upon by the three examiners, depending on the specific garment (see Supplementary Table 1 for a description of all patterns identified for each garment). Analysis of the dressing patterns displayed by healthy participants revealed a few common patterns, specific to each garment used in this study.

For all but two garments, more than one dressing pattern was identified (Table 1). For wearing gloves and trousers, all participants adopted a single dressing pattern. Each garment exhibited one prevalent dressing pattern, adopted by most participants (more than 50%), except for “wearing a scarf” (three patterns, each used by about 30% of participants) and “removing slippers” (one pattern at 50%, 3 other patterns used by less than 26% each).

**Table 1:** Distribution of the different dressing patterns in the healthy sample.

Garment wearing/removing	Number of patterns identified	% of healthy participants for each dressing/undressing pattern
Cap – wearing Cap – removing	3 4	89% pattern 1; 4% pattern 2; 7% pattern 3 73% pattern 1; 6% pattern 2; 3% pattern 3; 18% pattern 4
Gloves – wearing Gloves – removing	1 2	100% pattern 1 99% pattern 1; 1% pattern 2
Scarf – wearing Scarf – removing	4 4	30% pattern 1; 36% pattern 2; 3% pattern 3; 31% pattern 4 62% pattern 1; 4% pattern 2; 3% pattern 3; 31% pattern 4
T-shirt – wearing T-shirt – removing	3 3	24% pattern 1; 73% pattern 2; 3% pattern 3 69% pattern 1; 30% pattern 2; 1% pattern 3
Shirt – wearing Shirt – removing	2 3	71% pattern 1; 29% pattern 2 84% pattern 1; 6% pattern 2; 10% pattern 3
Cardigan – wearing Cardigan – removing	2 2	71% pattern 1; 29% pattern 2 96% pattern 1; 4% pattern 2
Zipper (cardigan) – zip up Zipper (cardigan) – unzip	2 2	77% pattern 1; 23% pattern 2 96% pattern 1; 4% pattern 2
Slippers – wearing Slippers – removing	2 4	93% pattern 1; 7% pattern 2 18% pattern 1; 26% pattern 2; 6% pattern 3; 50% pattern 4
Bathrobe – wearing Bathrobe – removing	2 3	70% pattern 1; 30% pattern 2 69% pattern 1; 4% pattern 2; 27% pattern 3
Trousers – wearing Trousers – removing	1 2	100% pattern 1 99% pattern 1; 1% pattern 2

#### 2.1.3 Statistical analysis

Firstly, binomial and multinomial logistic regressions were performed to examine whether the wearing patterns identified for each garment varied with the participant’s age and sex. Then, the relations between participants’ age and dressing/undressing execution times were explored using a series of correlation analyses with Spearman’s rank coefficient (*ρ*). We also tested whether execution times differed by participants’ sex using Mann–Whitney U tests.

All statistical analyses were performed using the software Jamovi (v. 2.7.5) (30), and statistical significance was set at *p* ≤ 0.05. For logistic regressions and *t*-tests, McFadden’s *R*^2^ and Cohen’s d were reported as effect size values, respectively.

#### 2.1.4 Results

Logistic regressions showed that the frequency of patterns changed as function of age for half of the evaluated items, namely “wearing the cardigan” (Z = -2.409, *p* = 0.016; model fit: χ^2^_1_ = 6.58, *p* = 0.01, *R*^2^ = 0.079), “wearing the shirt” (Z = -2.356, *p* = 0.018; model fit: χ^2^_1_ = 6.26, *p* = 0.012, *R*^2^ = 0.075), “wearing bathrobe” (Z = -2.507, *p* = 0.012; model fit: χ^2^_1_ = 7.18, *p* = 0.007, *R*^2^ = 0.084), “zip up the cardigan’s zipper” (Z = -3.047, *p* = 0.002; model fit: χ^2^_1_ = 12.41, *p* < 0.001, *R*^2^ = 0.165), and “removing the bathrobe” (model fit: χ^2^_2_ = 12.62, *p* = 0.002, *R*^2^ = 0.121). For all these “wearing” items, the older the participant, the lower the probability of using the second pattern (i.e., the less frequent one among the two identified – see Table 1). For “removing bathrobe”, the older the participant, the higher the probability of using the first pattern rather than the third (Z = -2.618, *p* = 0.009). No statistically significant regression models were found for the other garments (all χ^2^s < 7.24, all *p*s>0.065).

Considering participants’ sex, only “wearing the scarf” (Z = 2.39, *p* = 0.004; model fit: χ^2^_3_ = 11.72, *p* = 0.008, *R*^2^ = 0.07) changed in function of this factor. Females tend to wear the scarf using less the first pattern and more the second pattern than males. Other garments were not influenced by sex (all χ^2^s < 5.29, all *p*s>0.071).

**Figure 1:**
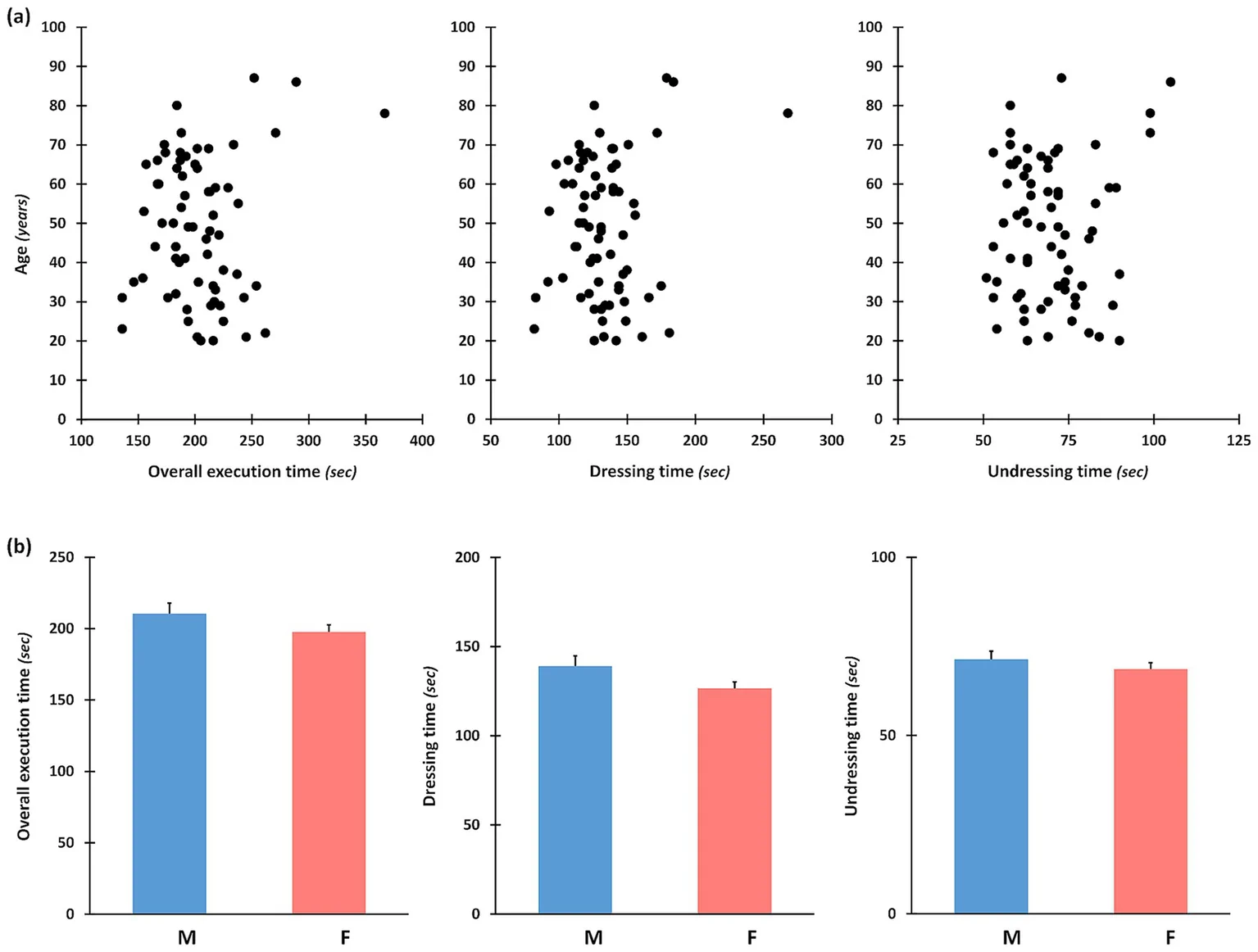
**(a)** Scatterplots between participants’ age and overall execution times (left panel), execution times for the dressing phase (central panel), and the undressing phase (right panel). No significant Spearman’s correlations were found. **(b)** Execution times according to participants’ sex (male = blue bars; female = red bars). Error bars = standard error. No significant differences were found.

Execution times for the ten garments correlated neither with the participants’ age, nor with overall performance (*ρ_68_* = −0.065, *p* = 0.595), nor with the dressing (*ρ_68_* = −0.029, *p* = 0.81) and undressing phases (*ρ_68_* = −0.052, *p* = 0.672) taken into account separately (Figure 1a). Similarly, there were no sex-specific differences in execution times (overall: U = 466.5, *p* = 0.114, *r* = 0.223; dressing phase: U = 447, *p* = 0.07, *r* = 0.255; undressing phase: U = 508.5, *p* = 0.28, *r* = 0.152; Figure 1b).

### 2.2 Study #2: assessment of dressing ability in brain-damaged patients: interrater reliability, clinical usability, and clinical differences

#### 2.2.1 Participants

Twenty-two unilateral brain-damaged patients (10 females, mean age ± SD: 69.7 ± 13.1 years, range: 45–87 years; mean education: 10.6 ± 4.1 years, range: 5–18 years) were recruited at the Neurorehabilitation unit of the IRCCS Istituto Auxologico Italiano in Milan (Italy). Exclusion criteria were a previous history or evidence of neurological (stroke, brain tumor, head injury, cerebral infections) and psychiatric disorders, the failure to understand the task, and/or no consent for video recording.

Twelve brain-damaged patients had a lesion affecting the left hemisphere (all with vascular etiology: 4 hemorrhagic, 8 ischemic), and ten had a lesion involving the right hemisphere (1 with neoplastic etiology, 9 with vascular etiology: 3 hemorrhagic, 6 ischemic). Average (±SD) length of illness (i.e., time elapsed since the stroke or since the surgical removal of the brain tumor) was 378.2 ± 1265.5 days (range: 7–5,840). Table 2 summarizes demographic traits and neurological characteristics of the 22 brain-damaged patients.

**Table 2:** Demographics and neurological profile of brain-damaged patients.

Patient no.	Sex	Age	Education (years)	Lesion side/Etiology	Length of illness (days)	NIHSS	FIM tot	FIM moto	FIM cog	CPM
P1	M	45	13	R/I	30	8	91	56	35	29
P2	M	58	13	R/H	44	11	77	47	30	32
P3	F	86	13	L/H	35	9	52	35	17	20
P4	F	61	11	R/I	33	7	101	70	31	23
P5	M	48	13	R/I	78	9	81	55	26	5
P6	F	66	18	R/I	198	3	85	55	30	/
P7	M	80	8	R/I	17	16	79	47	32	17
P8	M	82	18	L/I	100	13	59	26	33	13
P9	F	75	8	L/I	29	9	39	19	20	29
P10	M	77	18	L/I	1,060	10	67	35	32	32
P11	M	65	8	R/I	195	15	53	28	25	20
P12	M	77	9	L/I	26	2	80	58	22	12
P13	M	68	8	L/I	204	12	/	/	/	27
P14	F	45	8	R/N	5840	4	/	/	/	13
P15	M	73	8	L/H	34	5	62	31	31	24
P16	F	79	8	L/H	39	7	77	55	22	30
P17	F	62	13	L/I	22	4	54	42	12	25
P18	F	80	13	L/I	26	10	46	36	10	31
P19	M	64	5	L/I	75	13	37	22	15	14
P20	F	87	5	R/H	7	14	35	17	18	23
P21	M	78	18	L/I	880	4	/	/	/	10
P22	F	85	5	R/I	30	8	91	58	33	12

R, right-sided lesion; L, left-sided lesion; I, ischemic stroke; H, hemorrhagic stroke; N, neoplastic lesion; NIHSS, National Institute of Health Stroke Scale; FIM, Functional Independence Measure; FIM moto, scoring at FIM items assessing motor functions; FIM cog, scoring at FIM items assessing cognitive functions; CPM, Raven’s Colored Progressive Matrices.

Before the experiment, all patients were administered the Mini-Mental State Examination (31, 32) and the Raven’s Colored Progressive Matrices [CPM; Basso et al. (33)]. Permission to use the MMSE in our research was granted by buying a set of licenses from the Italian licensing partner. Left-brain-damaged patients were given standardized neuropsychological tests to assess the presence of aphasia (34) and apraxia: constructional (21), ideomotor (18), ideational (19), and buccofacial (21). In right brain-damaged patients, the presence of unilateral spatial neglect (USN) was assessed by administering the Bell Cancelation test (35), the Five Elements Complex Drawing Test (36), the Clock drawing test (37), and the Fluff test (38).

The FIM (2), an 18-item assessment tool, was also administered to evaluate patients’ level of independence in performing activities of daily living (18–126; the higher the score, the greater the patient’s functional independence). The 11-item version of the National Institutes of Health Stroke Scale [NIHSS; Kwah and Diong (39)], a scale for assessing stroke severity (0–42; higher scores indicate more severe stroke), was also administered.

#### 2.2.2 Experimental procedure

All brain-damaged patients were asked to wear and remove the 8 out of 10 garments used for the healthy participants: cap, gloves, scarf, T-shirt, shirt, cardigan, zipper cardigan, and slippers. Since the majority of them could not wear trousers and a bathrobe, mainly due to the motor deficits that prevented the test from being performed safely, these two items were not administered to patients.

Firstly, the examiner checked that the instructions were fully understood, providing clarification as needed to ensure that all patients, especially those with language deficits, could understand them.

Patients were asked to wear the garment displayed in front of them and then to remove it. For the zipper cardigan, patients had to zip and unzip it. Patients were video-recorded during the task. Videos were evaluated by the same three independent raters who participated in *Study #1* (I.R., A.R., C.C.), blinded to one another’s scores. An ad-hoc evaluation grid was prepared to record dressing skills and variations in dressing patterns observed among healthy participants during the first phase of the study. After the assessment, in the event of disagreement among the three raters, the exact item score to be assigned was resolved by consensus among all raters, taking into account the different ratings.

A 3-point rating scale was developed to assess the presence of a dressing disorder and functional independence in dressing/undressing the 8 garments used in the test, balancing clinical sensitivity with inter-rater reliability by explicitly accounting for both qualitative praxic errors and functional autonomy. The scoring was defined as follows:

- Score = 0, the patient showed a disorder of dressing, namely, they did not adopt any of the patterns of dressing identified in healthy participants (regardless of upper- and lower-limb motor impairments), and is entirely dependent in dressing/undressing.
- Score = 1. The patient showed a deficit of dressing, as defined above, but with residual autonomy in dressing/undressing.
- Score = 2. The patient did not show a deficit of dressing, namely: he or she was fully autonomous in dressing/undressing, and used the repertoire of dressing patterns identified in healthy participants.

Therefore, the total score on the MIDA test ranged from 0 to 16. A patient was considered to show a deficit of dressing when they did not use at least one of the dressing patterns of healthy participants, namely: the patient’s score ranged from 0 to 15, depending on the degree of functional independence in wearing the various clothing items; lower scores indicated severe disorder with complete dependence in dressing. However, it is important to note that this operational definition of dressing impairment is provisional, serving as a preliminary metric to capture behavioral variations in dressing. Indeed, a dressing strategy not observed in the healthy sample does not automatically imply a pathological performance since it may reflect, for instance, functional compensation for hemiparesis, individual habits, handedness, specific garment characteristics, or cultural variability.

Notably, the total execution time taken to complete the tasks was not included as a score for the clinical sample. In the brain-damaged population, execution speed is heavily confounded by elementary motor deficits (e.g., hemiplegia or hemiparesis). Omitting time measurements ensured that the MIDA remained uniquely sensitive to praxic errors, rather than generic motor slowdowns.

The entire testing session, including instructions and full administration of the MIDA, took approximately 15–20 min, with scoring completed during the test itself.

The MIDA scoring sheet/grid is freely available in the Supplementary Table 1.

To note: video recording was employed in this initial study to facilitate a detailed analysis of inter-rater reliability, but it is not required for the clinical application of the test. Clinicians can directly score the patient in real time using the provided MIDA scoring sheet (see Supplementary materials 1: MIDA Scoring sheet/grid).

#### 2.2.3 Statistical analysis

Firstly, we assessed whether patients’ total scores on the MIDA showed high agreement (i.e., interrater reliability) among our three raters using the intraclass correlation coefficient (ICC – model: two-way random; type: single measurement; definition: agreement, i.e., ICC 2,1) (40). As a rule of thumb, ICC values less than 0.5, between 0.5 and 0.75, between 0.75 and 0.9, and greater than 0.9 indicate poor, moderate, good, and excellent reliability, respectively (41).

Then, to better define the clinical value of our scale, we assessed whether the MIDA total score correlated with the NIHSS, FIM total score, and the mean score at the two FIM items assessing dressing autonomy (i.e., FIM items 4 and 5, evaluating dressing autonomy for upper and lower body garments, respectively). As reported in Table 2, FIM and CPM data were unavailable for three (P13, P14, and P21) and one patient (P6), respectively, due to transfer or discharge before completion of the clinical evaluation. These missing cases were handled via listwise exclusion on an analysis-by-analysis basis.

We also explored, using Spearman’s correlations, possible associations between MIDA score and patients’ age, education level, length of illness, and non-verbal reasoning (i.e., CPM scores).

Finally, Mann–Whitney U tests were used to explore whether differences in lesion side and the presence/absence of neuropsychological deficits were associated with MIDA scores, thereby influencing dressing skills.

All statistical analyses were performed using the software Jamovi [v. 2.7.5, (30)]. ICC analyses were performed with Jamovi’s SimplyAgree module (42). Statistical significance was set at *p* ≤ 0.05. For Mann–Whitney tests, the rank-biserial correlation (r) was reported as the effect size.

#### 2.2.4 Results

Overall, 12 out of 22 brain-damaged patients (55%) showed no signs of a dressing disorder (i.e., MIDA total score = 16/16), as they displayed the same dressing patterns observed in healthy participants (see Section 2.1 and Supplementary Table 1) and could put on each garment quickly, effectively, and unassisted. The remaining 10 brain-damaged patients (45%) obtained a mean MIDA score of 9.4/16 (range = 2–15), showing anomalies in dressing patterns for at least one garment.

MIDA’s interrater reliability, and hence raters’ agreement, was excellent, with the total scale score yielding an ICC of 0.934 (confidence intervals: 0.885–0.965). ICCs for the single garments were presented in Supplementary Table 2: they all present moderate to excellent reliability (range: 0.536–0.963).

**Figure 2:**
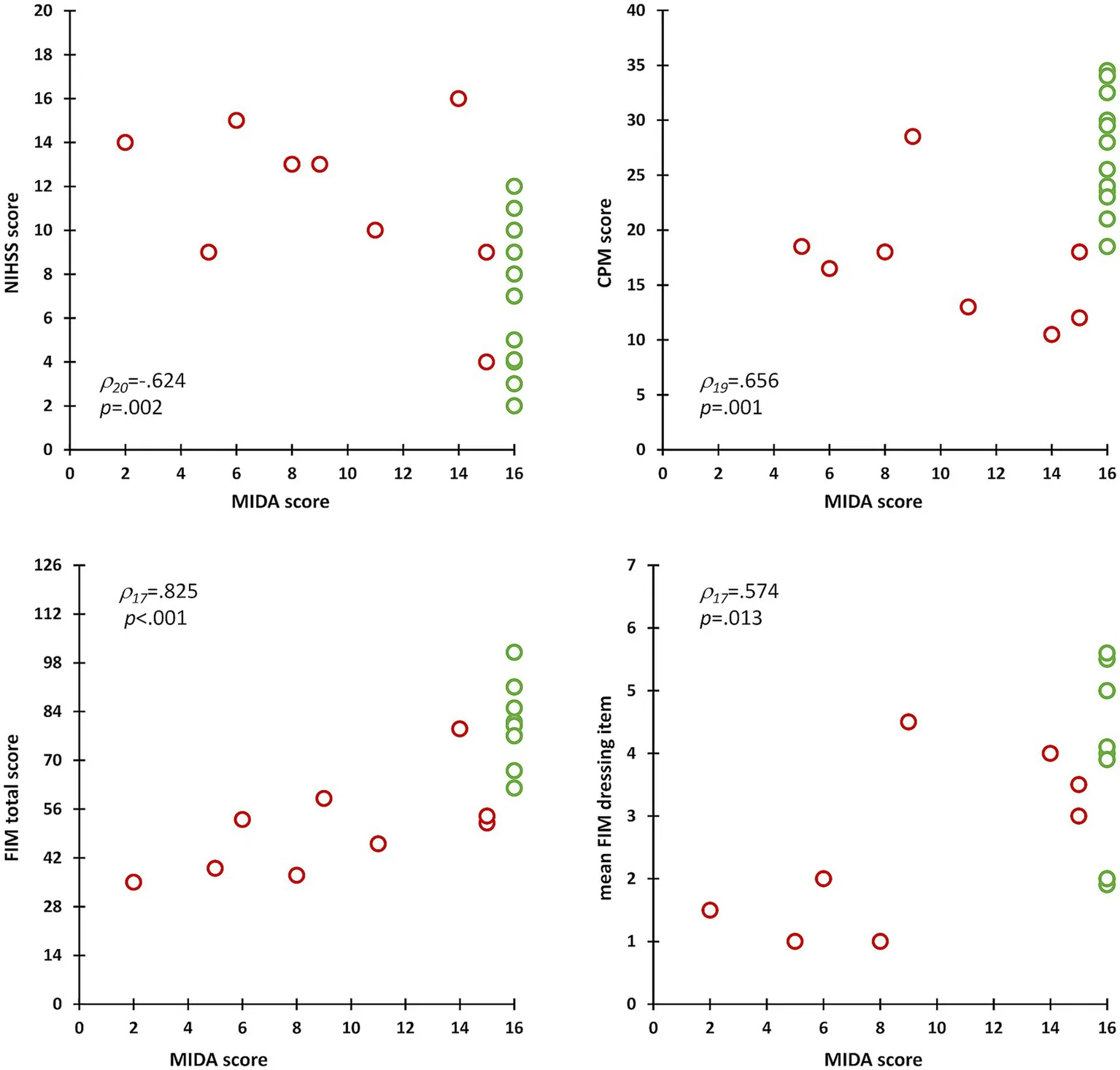
Scatterplots illustrating the relationships between MIDA scores and clinical variables in brain-damaged patients. From left to right and up to down: NIHSS scores, CPM scores, FIM total scores, and FIM dressing item scores. MIDA scores showed statistically significant Spearman’s correlations with all variables. Green dots indicate patients without dressing disorders (i.e., MIDA score = 16), red dots indicate patients with dressing disorders (i.e., MIDA score ≤15).

MIDA scores significantly correlated with NIHSS (*ρ_20_* = −0.624, *p* = 0.002) and FIM (*ρ_17_* = 0.825, *p* < 0.001) scores, as well as with the mean score at the FIM items assessing dressing autonomy (*ρ_17_* = 0.574, *p* = 0.013) and with CPM scores (*ρ_19_* = 0.656, *p* = 0.001, Figure 2). At the same time, the MIDA did not correlate with participants’ age (*ρ_20_* = −0.37, *p* = 0.09), educational level (*ρ_20_* = 0.209, *p* = 0.351), and duration of disease (*ρ_20_* = 0.389, *p* = 0.074).

**Figure 3:**
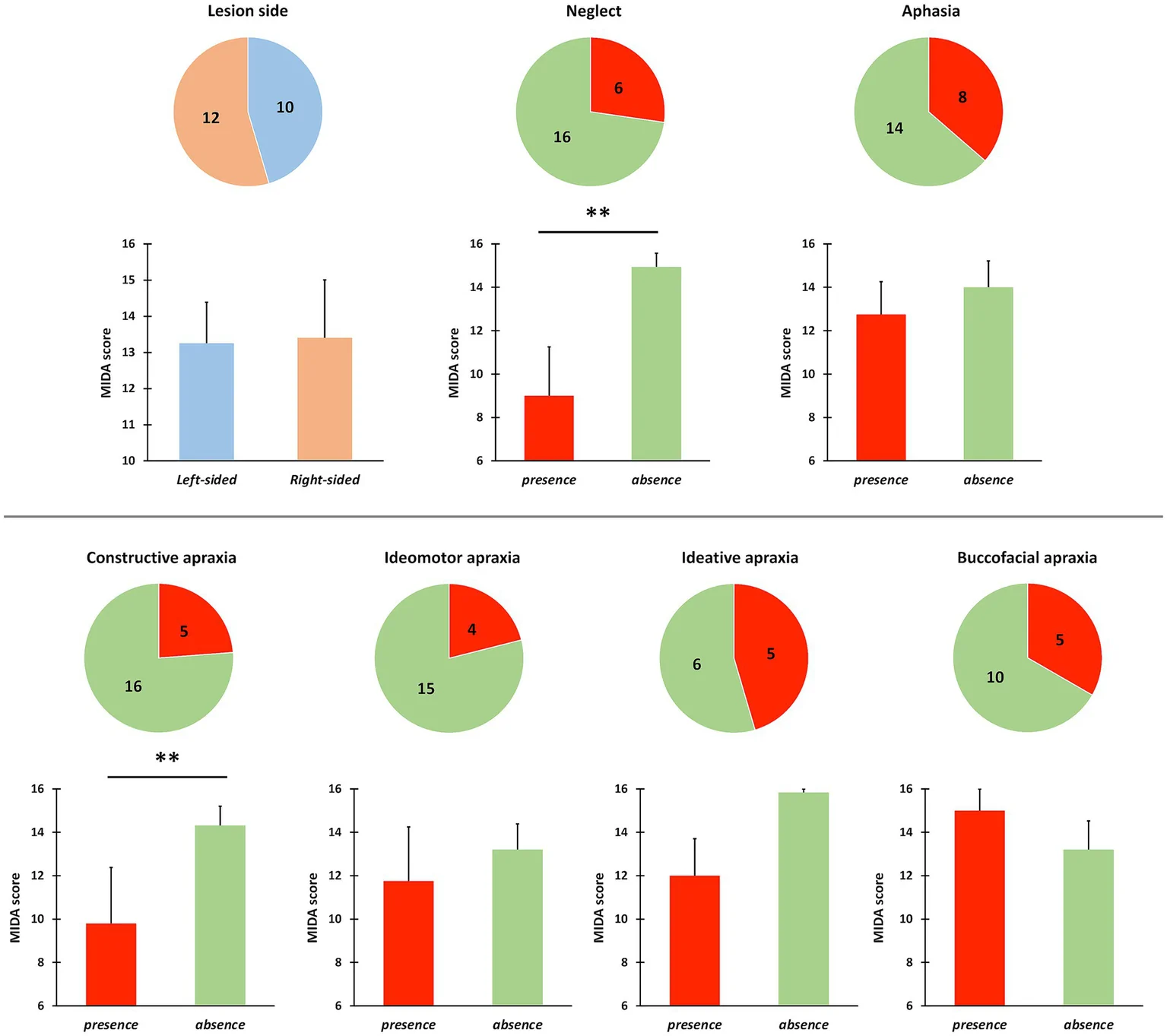
MIDA scores in brain-damaged patients according to lesion side (blue bars and segments = left-sided lesion, orange bars and segments = right-sided lesion) and the presence (red bars and segments) or absence (green bars and segments) of a neuropsychological deficit. Significant differences in MIDA scores were found for neglect and constructional apraxia. The pie charts show the number of brain-damaged patients in each group (as stated in Section 2.2.1; not all patients underwent the same neuropsychological battery, which was tailored to the lesion’s hemispheric location). Error bars = SE. * = *p* < 0.05; ** *p* < 0.01.

Furthermore, no statistically significant differences in MIDA score were found between left and right brain-damaged patients (U = 52, *p* = 0.579, *r* = 0.133, Figure 3, first panel).

Considering the neuropsychological deficits (Figure 3), significant differences in MIDA scores were found according to the presence of neglect (U = 14, *p* = 0.006, *r* = −0.708; patients with neglect – mean ± ES: 9 ± 2.25, patients without neglect: 14.94 ± 0.64) and constructional apraxia (U = 17, *p* = 0.033, *r* = −0.575; patients with constructional apraxia: 9.8 ± 2.58, patients without constructional apraxia: 14.31 ± 0.89). No differences were found for patients with aphasia (U = 41, *p* = 0.267, *r* = −0.268), ideomotor (U = 21.5, *p* = 0.301, *r* = −0.328), ideational (U = 7, *p* = 0.113, *r* = −0.533), and buccofacial apraxia (U = 17, *p* = 0.3, *r* = 0.32).

## 3 Discussion

*Study #1* investigated dressing skills in healthy individuals to identify the patterns used for different garments. Such patterns were subsequently used to assess dressing skills in brain-damaged patients (*Study #2*). This allowed us to propose a novel, preliminary clinical instrument for the evaluation of dressing deficits: the MIlan Dressing Assessment (MIDA).

### 3.1 Dressing abilities in healthy individuals

Dressing skills are acquired during childhood, but, to the best of our knowledge, the patterns of this complex behavior have never been systematically described in healthy participants. Clegg et al. (43) identified a set of primitive actions that are practical for human dressing; however, the various dressing actions were described as isolated, rather than grouped into patterns. This investigation is therefore not suitable for the clinical assessment of dressing deficits (43).

Conversely, in the present two studies, the possible ways of dressing or undressing different garments were specifically investigated. We found that some garments can be weared or taken off in various ways: three/four different patterns were used to dress or undress caps, scarfs, T-shirts, shirts, slippers and bathrobes, while other clothes (gloves, trousers) were put on/off in just one/two ways, as also the cardigan button and zipper can be opened or closed in just two ways. Notably, the participants’ biological sex does not influence dressing patterns and their execution times.

Notably, as age increases, no new or different patterns are observed. Indeed, for all “wearing” items, the choice of the most frequent dressing/undressing pattern (i.e., pattern 1 in Table 2) dominates across ages, while the probability of using other ways of dressing/undressing decreases. This suggests that the fundamental repertoire of behaviors related to dressing/undressing remains stable over time, i.e., the same dressing patterns are found between the ages of 20 and 87 (the age range of this experimental sample). Still, as people age, behavior becomes less flexible, and one behavioral mode tends to dominate.

On the one hand, age makes healthy people less flexible in their dress; on the other hand, it does not affect how quickly they dress. Despite the sensorimotor and cognitive slowdown that characterizes older adults (44, 45), this does not impact dressing patterns, suggesting that dressing is a complex, multi-step, largely automated behavior and therefore less susceptible to the age-related psychophysical decline.

### 3.2 Dressing abilities in brain-damaged patients and their assessment with the MIDA

Studies of individual cases mainly characterize the neuropsychological literature on disorders of dressing, specifically dressing apraxia (46–48). The present study is the first to investigate dressing behavior in a sample of patients with acquired right- and left-sided hemispheric lesions, using a test with norms based on the performance of healthy participants. The high interrater reliability among the three independent raters underscores the reliability of the proposed evaluation procedure in brain-damaged patients.

Using the patterns of dressing and undressing for the eight garments used by healthy persons as a reference, we found that almost 45% of brain-damaged patients, regardless of lesion side, had difficulty dressing and failed to dress successfully with typical strategies. The remaining patients achieved maximum scores, resulting in a noticeable ceiling effect. However, given that more than half of our brain-damaged sample did not present with clinically overt dressing apraxia, their flawless execution and consequent maximum scores are entirely expected. This demonstrates that the MIDA does not artificially penalize patients with diffuse brain damage; rather, it enables clinicians to detect genuine dressing impairments, thereby confirming its utility in distinguishing spared from impaired performance.

The severity of the dressing disorder, as reflected by the total MIDA score, co-varies with overall clinical and functional status. Specifically, it is negatively associated with the NIHHS score, indicating that dressing abilities decrease as neurological severity increases, but positively correlated with the total FIM score, indicating that a more severe dressing impairment reflects a lower degree of the patient’s global functional independence. Crucially, we evaluated concurrent validity by analyzing the relationship between MIDA scores and an established functional measure, namely the FIM dressing item. Although a strong positive association emerged—providing preliminary evidence of concurrent validity, as higher MIDA scores align with greater independence on the FIM dressing item—this correlation must be interpreted with caution. Indeed, because both tools inherently evaluate functional autonomy, this association is partly expected due to conceptual overlap and should not be considered an indicator of broader clinical reliability.

Finally, the severity of the inability to dress oneself, as reflected in the MIDA scores, is associated with reasoning abilities (CPM score), confirming the dependence of dressing capacity on visuospatial problem-solving (46, 49).

In this study, left- and right-brain-damaged patients present with a similar level of dressing disabilities. Given our small sample size, the comparisons involving specific clinical subgroups (such as those based on lesion side, neglect, aphasia, or limb apraxia) must be considered purely exploratory and interpreted with caution, as they carry an inherent risk of statistical instability or false-negative findings. Nevertheless, these preliminary analyses provide valuable insights that align with established literature. For instance, the combination of neglect and constructional apraxia makes difficulty dressing more likely, in line with previous evidence (46, 50–53), likely due to shared visuomotor or visuospatial deficits (54). We further observed that dressing skills deficits, as reflected by the MIDA score, were not different in patients having ideomotor, ideative, or buccofacial apraxia (compared to patients without these disorders), supporting the hypothesis that the different forms of apraxia reflect distinct pathological mechanisms related to motor planning of actions (11). However, fine components of dressing, such as buttoning, have been shown to be strictly dependent on distinct praxic dimensions, specifically limb-kinetic apraxia, which significantly impacts functional autonomy regardless of elementary motor impairments (55).

### 3.3 The MIDA

This study explores dressing patterns in healthy individuals to outline reference evaluative models, investigates them in a clinical sample of brain-damaged patients, and proposes a novel structured framework for the preliminary evaluation of these skills. In this view, the MIDA is primarily designed as a quantitative scale of execution patterns and residual autonomy in dressing, rather than a qualitative error checklist or a formal error taxonomy. In a translational perspective, focusing on garment-specific dressing patterns and the patient’s residual independence in dressing tasks could offer valuable, actionable information regarding what the patient can actually do. Indeed, time-honored observational protocols (23) and more recent instruments (26) have advanced the detection of post-stroke dressing deficits, although focusing only on upper-body dressing. Conversely, the MIDA addresses this limitation by covering multiple body parts with a comprehensive set of distinct garments. Furthermore, by leveraging normative data from healthy individuals, the approach is designed to help clinicians to explore and characterize potential alterations in behavioral dressing patterns. Alongside these clinical features, a key practical advantage of the MIDA lies in its brief administration and scoring time, typically requiring about 20 min. These features suggest that the MIDA holds promise for routine clinical assessment of dressing apraxia, although formal feasibility and validation studies are required to fully confirm its diagnostic utility.

To facilitate its adoption, the complete administration and scoring protocol, including the generation of an automatic graph to visualize patients’ performance, is available in the Supplementary materials.

### 3.4 Limitations

Yet, the present, exploratory study has some limitations. First, the size of the sample of brain-damaged is relatively small, and only some of them showed dressing apraxia. This reduces the overall statistical power for broader generalization. Consequently, the exploratory comparisons among clinical subgroups (e.g., regarding neglect, aphasia, or lesion side) may suffer from limited statistical power. Additionally, brain-damaged participants were recruited from a single rehabilitation center, while healthy participants were enrolled through convenience sampling, potentially introducing selection bias. Furthermore, test–retest reliability was not assessed due to logistical constraints during the rehabilitation stay. Evaluating the performance stability over time, as well as further investigating the construct validity of the MID, represents a necessary next step. Future standardization on larger and more diverse clinical cohorts will be essential to fully confirm the MIDA’s psychometric robustness.

## Data Availability

The datasets presented in this study can be found in online repositories. The names of the repository/repositories and accession number(s) can be found at: Open Science Framework (https://osf.io/2ebcr/) upon manuscript acceptance. Further information is available from the corresponding authors upon reasonable request.
