# Supplementary material for: Dressing patterns in healthy individuals and brain-damaged patients: the MIlan Dressing Assessment
Source: Front Neurol. 2026 Aug 12;17:1852682. doi: 10.3389/fneur.2026.1852682 (PMC13506407; doi:10.3389/fneur.2026.1852682)
Supplement: Supplementary file 1 [file Data_Sheet_1.PDF]

MIDA: Scoring sheet and instructions.

THE MILAN DRESSING ASSESSMENT – MIDA<br>SCORING SHEET			
**GARMENT**	**SCORE**	**PUT ON**	**TAKE OFF**
**Cap**	0 – 1 – 2	Pattern 1: Block Front, Pull Down Back<br>Pattern 2: Top-down<br>Pattern 3: Hook Back, Adjust Front	Pattern 1: Pull from the Tip<br>Pattern 2: Lateral Pull<br>Pattern 3: Pull from Back, Take Off Front<br>Pattern 4: Pinch/Palm on Front, Pull Off Back
**Gloves**	0 – 1 – 2	Pattern 1: Fingers First<br>Pattern 2: No Hand Assistance	Pattern 1: Pinch and Pull<br>Pattern 2: Pull Down from the Wrist<br>Pattern 3: Pinch Fingers Together and Pull Off
**Scarf**	0 – 1 – 2	Pattern 1: Folded over Shoulders<br>Pattern 2: Wrap around the Neck	Pattern 1: Take Off from One Side<br>Pattern 2: Unfasten and Take Off
**Shirt**	0 – 1 – 2	Pattern 1: One Arm First<br>Pattern 2: Arms First, then Head	Pattern 1: One Arm Out, then the Other<br>Pattern 2: With Both Hands at the Back of the Neck
**T-Shirt**	0 – 1 – 2	Pattern 1: Head First, then Arms<br><br>Pattern 2: Arms First, then Head	Pattern 1: Pull from Collar, Head First<br>Pattern 2: Pull from Waist, Head Last<br>Pattern 3: Assisted Takeoff
**Cardigan**	0 – 1 – 2	Pattern 1: One Arm, then the Other<br>Pattern 2: Put it on the Shoulders	Pattern 1: One Arm Out, then the Other<br>Pattern 2: Pull it from the Neck
**Zipper (Cardigan)**	0 – 1 – 2	Pattern 1: Hand on the Zipper<br>Pattern 2: One Hand Holds the Garment	Pattern 1: Pull Down<br>Pattern 2: Pull Down from the Bottom
**Slippers**	0 – 1 – 2	Pattern 1: No Hand Assistance<br>Pattern 2: With Hand Assistance	Pattern 1: Remove One Foot Alone, Then Block the Other<br>Pattern 2: Both Feet Block Each Other<br><br>Pattern 3: Use of Hands<br>Pattern 4: No Hand or Feet Assistance
**TOTAL SCORE_____/16**			

Scoring:
2 = The patient uses one of the physiological patterns.
1 = The patient does not use one of the physiological patterns but can put on/take off the garment independently.
0 = The patient does not use the physiological patterns and is unable to put on/take off the garment.

ADMINISTRATION AND SCORING INSTRUCTIONS

The **Milan Dressing Assessment (MIDA)** is a 15-minute clinical tool designed to evaluate dressing impairments in brain-damaged patients by comparing their behaviors against established physiological dressing patterns.

1. Test Administration Overview

- **Target Garments**: The assessment evaluates 8 specific garments suited for different body parts: cap, gloves, scarf, T-shirt, shirt, cardigan, zipper cardigan (zipping/unzipping), and slippers.
- **Method**: Observe the patient putting on and taking off each garment naturally. Real-time scoring is conducted directly by using the scoring sheet.

2. The 3-Point Scoring System

For each of the 8 garments, assign a score of 0, 1, or 2 based on the patient's performance. The score must reflect a balance between qualitative praxic execution (whether they use physiological patterns) and functional independence:

- **Score 2 (Normal Dressing Pattern)**:
  - The patient is fully autonomous in putting on and taking off the garment.
  - The patient successfully employs one of the physiological dressing patterns identified in healthy controls (refer to the MIDA Scoring Sheet for specific patterns per garment).
- **Score 1 (Impaired Pattern with Residual Autonomy)**:
  - The patient presents a dressing deficit, meaning they do not adopt any of the physiological patterns identified in healthy individuals.
  - However, the patient exhibits residual autonomy and is still able to successfully put on and take off the garment independently using alternative or compensatory strategies.
- **Score 0 (Impaired Pattern with Dependence)**:
  - The patient presents a dressing deficit, failing to use any physiological dressing patterns.
  - The patient is unable to put on or take off the garment and is entirely dependent on the examiner for assistance.

3. Total Score and Interpretation

- **Total Score Range**: **0 to 16** (calculated by summing the scores of all 8 garments).
- **Score of 16**: Indicates **no dressing disorder** (the patient's dressing patterns align with healthy individuals, and they are fully independent).
- **Score of 15 or less**: Indicates the presence of **dressing apraxia / dressing impairment**. Lower scores represent more severe apraxic deficits and complete dependence in dressing.
