# Supplementary material for: Dressing patterns in healthy individuals and brain-damaged patients: the MIlan Dressing Assessment
Source: Front Neurol. 2026 Aug 12;17:1852682. doi: 10.3389/fneur.2026.1852682 (PMC13506407; doi:10.3389/fneur.2026.1852682)
Supplement: Supplementary file 2 [file Table_1.DOCX]

### Supplemental Table 1.

| **PATTERNS USED BY HEALTHY SUBJECTS IN STUDY #1, BY GARMENT** | | | |
| --- | --- | --- | --- |
| **Garment** | **Phase** | **Pattern** | **Description** |
| **CAP** | **Put On** | Pattern 1: Block Front, Pull Down Back | The subject takes the back edge of the cap with both hands, spreads it, blocks it in front of the forehead, and pulls it down at the back. |
|  |  | Pattern 2: Top-down | The subject takes the lateral edge of the cap with both hands, spreads it, and places it from the top at the center of the head, adjusting the sides and back as needed. |
|  |  | Pattern 3: Hook Back, Adjust Front | The subject takes the back edge of the cap with both hands, spreads it, holds the cap at the nape, and pulls it down in front. |
|  | **Take Off** | Pattern 1: Pull from the Tip | One hand takes the tip of the cap and pulls it off from the front. |
|  |  | Pattern 2: Lateral Pull | One hand takes the cap laterally and pulls it off by rotating it forward. |
|  |  | Pattern 3: Pull from Back, Take Off Front | With both hands, the subject takes the cap from the nape, lifts their hands over the head, and pulls it off from the front. |
|  |  | Pattern 4: Pinch/Palm on Front, Pull Off Back | With one hand, the subject pinches or palms the front of the cap and pulls it off from the back. |
| **GLOVES** | **Put On** | Pattern 1: Fingers First | The subject slides the fingers in and pulls the glove with the thumb, then adjusts it with the other hand. |
|  |  | Pattern 2: No Hand Assistance | The subject uses the thumb to insert the other thumb in the respective hole. |
|  | **Take Off** | Pattern 1: Pinch and Pull | The subject pinches the glove from the tip of a finger and pulls it off. |
|  |  | Pattern 2: Pull Down from the Wrist | The subject uses the fingers of the other hand to pull down the glove from the wrist. |
|  |  | Pattern 3: Pinch Fingers Together and Pull Off | The subject uses the thumb and index finger to pull the glove off by rolling it up. |
| **SCARF** | **Put On** | Pattern 1: Folded over Shoulders | The subject places the scarf folded over the shoulders and fixes it in front. |
|  |  | Pattern 2: Wrap around the Neck | The subject places the scarf on one shoulder and wraps it around the neck with a single movement. |
|  | **Take Off** | Pattern 1: Take Off from One Side | The subject takes the end of the scarf from one side and pulls it off completely. |
|  |  | Pattern 2: Unfasten and Take Off | The subject unfastens the scarf with a movement and pulls it away from the neck. |
| **SHIRT** | **Put On** | Pattern 1: One Arm First | The subject takes one hand, puts it into the sleeve from the front, passes the shirt behind the back to find the other sleeve, and finally inserts the other hand. |
|  |  | Pattern 2: Arms First, then Head | The subject puts both arms into the respective sleeves from the back and then pulls the shirt over their shoulders. |
|  | **Take Off** | Pattern 1: One Arm Out, then the Other | The subject takes one arm out of the sleeve, pulls the shirt off the cap side, and then removes the shirt from the other arm. |
|  |  | Pattern 2: With Both Hands at the Back of the Neck | The subject takes the shirt from the back of the neck and pulls it off. |
| **T-SHIRT** | **Put On** | Pattern 1: Head First, then Arms | The subject takes the t-shirt from the collar with both hands, puts it over their head, then slips each sleeve into one arm, followed by the other. |
|  |  | Pattern 2: Arms First, then Head | The subject puts both arms into the sleeves from the back, takes the t-shirt up the back with both hands, lifts it over the head, and pulls it down. |
|  | **Take Off** | Pattern 1: Pull from Collar, Head First | The subject takes the collar with hands crossed, pulls the t-shirt over the head, and then takes out one arm, followed by the other. |
|  |  | Pattern 2: Pull from Waist, Head Last | The subject takes the hem of the t-shirt with both hands, pulls it up to the armpits, helps each arm exit the sleeves, and finally lifts the t-shirt over the head. |
|  |  | Pattern 3: Assisted Takeoff | The subject's hands cross in front to take the back of the t-shirt, pulls it upward, and one arm bends and exits the sleeve as the t-shirt slips off the head. |
| **CARDIGAN** | **Put On** | Pattern 1: One Arm, then the Other | The subject puts one arm in the sleeve, then the other. |
|  |  | Pattern 2: Put it on the Shoulders | The subject places the cardigan on the shoulders and puts the arms into the sleeves. |
|  | **Take Off** | Pattern 1: One Arm Out, then the Other | The subject takes one arm out of the sleeve, and then the other. |
|  |  | Pattern 2: Pull it from the Neck | The subject takes the cardigan from the neck and pulls it off. |
| **ZIPPER (CARDIGAN)** | **Zip Up** | Pattern 1: Hand on the Zipper | The subject holds the zipper with one hand and zips it up. |
|  |  | Pattern 2: One Hand Holds the Garment | The subject holds the bottom of the garment with one hand and zips it up with the other. |
|  | **Unzip** | Pattern 1: Pull Down | The subject pulls the zipper down to unzip it. |
|  |  | Pattern 2: Pull Down from the Bottom | The subject holds the garment with one hand and unzips it from the bottom. |
| **SLIPPERS** | **Put On** | Pattern 1: No Hand Assistance | The subject slips feet into the respective slippers without using hands. |
|  |  | Pattern 2: With Hand Assistance | The subject uses their hands to slip into the slippers. |
|  | **Take Off** | Pattern 1: Remove One Foot Alone, Then Block the Other | One subject’s foot slides out of the slipper, holds the other slipper in place, and the foot slips out. |
|  |  | Pattern 2: Both Feet Block Each Other | One subject’s foot blocks the heel of the opposite slipper with its toes, the foot slips out, and the removed foot blocks the heel of the opposite slipper with its toes to remove it. |
|  |  | Pattern 3: Use of Hands | The subject uses the hands to pull the slippers off from the toes while the feet pull back. |
|  |  | Pattern 4: No Hand or Feet Assistance | The subject slips the slippers off by pulling the foot back. |
| **BATHROBE** | **Put On** | Pattern 1: First Sleeve On, Then The Other Arm Bends Behind The Back | The arm without a sleeve bends behind the back (or raises high above the head) to grab the free sleeve. The arm slides into the sleeve. The person raises their shoulders, rotating them forward to "hook" the bathrobe, and bends the torso forward to facilitate shoulder movement. The hands take hold of the respective edges of the bathrobe and bring the ends together. With both hands (each on the respective edge of the bathrobe), close the bathrobe. |
|  |  | Pattern 2: Arm Rotation (Garment "Flies" Behind The Back) | Takes the bathrobe with both hands from the shoulders. Rotates the bathrobe behind the back (pivoting on the shoulder). The arms slide into the respective sleeves, one at a time. The person bends the torso forward to facilitate shoulder movement. The hands take hold of the respective edges of the bathrobe and bring the ends together. |
|  | **Take Off** | Pattern 1: Sleeves Slide Backward, One Hand Holds The Opposite Sleeve | Both hands take the respective ends of the bathrobe and open it outwards. The arms bend behind the back to slide the bathrobe towards the wrists. One hand holds the opposite sleeve still so that the opposite arm can slip out. Both arms are brought forward to the front of the torso. The free hand pulls the sleeve off the opposite arm, which helps by pulling back. |
|  |  | Pattern 2: Arms Slip Out Of Sleeves From The Front | Both hands take the respective ends of the bathrobe and open it outwards. Bring both arms forward (with sleeves at wrist level). One hand holds the opposite sleeve so that the opposite arm can slip out by pulling back. Once free, the arm is brought forward. The free hand pulls the sleeve off the opposite arm, which helps by pulling back. |
|  |  | Pattern 3: Sleeves Slide From The Back Without Aid Of The Other Arm | Both hands take the respective ends of the bathrobe and open it outwards. Both arms bend behind the back to slide the bathrobe towards the wrists. Without needing the other hand, one arm slides out of the sleeve. Bring both arms forward. The free arm pulls the opposite sleeve forward while the opposite arm pulls back to slip out. |
| **TROUSERS** | **Put On** | Pattern 1 | Both hands hook the respective upper edges of the pants (at hip level). The arms "toss" the pants forward to straighten them. One leg bends and slips into the pants (weight on the opposite leg). The arms pull the pants upwards, **and then the subject repeats** the same with the other leg. Both hands hook the waistband of the pants and pull them up. |
|  | **Take Off** | Pattern 1: With Hands | Both hands hook the respective edges of the pants (at the hips) and pull them down. Once at knee level, one leg bends. While the leg is raised, the opposite hand helps by pulling the pant leg downwards. Once removed, the weight shifts to the opposite leg, and the hand pulls the pant leg off from the bottom (from the foot). |
|  |  | Pattern 2: Without Hands | Both hands hook the respective edges of the pants (at the sides, near the hips) and pull them down. Once at knee level, one leg bends. Without the help of the hands, alternately lift the right and left leg to make the pants drop (like "marching"). Until one leg comes out of the pants. Then the foot holds the opposite pant leg still so that the opposite foot can also slip out. |

### Supplemental Table 2. Interrater reliability for the eight garments of the MIDA, assessed with the ICC (model: two-way random; type: single measurement; definition: agreement).

| **Garments** | **ICC (2,1)** | **Confidence interval (95%)** |
| --- | --- | --- |
| *Cap* | .663 | .485 - .806 |
| *Gloves* | .929 | .877 - .963 |
| *Scarf* | .958 | .927 - .978 |
| *Shirt* | .865 | .768-.93 |
| *T-shirt* | .731 | .577 - .849 |
| *Zipper cardigan* | .776 | .636 - .878 |
| *Button cardigan* | .963 | .934 - .981 |
| *Slippers* | .536 | .32 - .747 |
